# Semi–Selective Fatty Acyl Reductases from Four Heliothine Moths Influence the Specific Pheromone Composition

**DOI:** 10.1371/journal.pone.0037230

**Published:** 2012-05-16

**Authors:** Åsa K. Hagström, Marjorie A. Liénard, Astrid T. Groot, Erik Hedenström, Christer Löfstedt

**Affiliations:** 1 Pheromone group, Department of Biology, Lund University, Lund, Sweden; 2 Institute for Biodiversity and Ecosystem Dynamics, University of Amsterdam, Amsterdam, The Netherlands; 3 Department of Entomology, Max Planck Institute for Chemical Ecology, Jena, Germany; 4 Department of Natural Sciences, Engineering and Mathematics, Mid Sweden University, Sundsvall, Sweden; INRA-UPMC, France

## Abstract

**Background:**

Sex pheromones are essential in moth mate communication. Information on pheromone biosynthetic genes and enzymes is needed to comprehend the mechanisms that contribute to specificity of pheromone signals. Most heliothine moths use sex pheromones with (*Z*)–11–hexadecenal as the major component in combination with minor fatty aldehydes and alcohols. In this study we focus on four closely related species, *Heliothis virescens*, *Heliothis subflexa*, *Helicoverpa armigera* and *Helicoverpa assulta*, which use (*Z*)–11–hexadecenal, (*Z*)–9–tetradecanal, and (*Z*)–9–hexadecenal in different ratios in their pheromone blend. The components are produced from saturated fatty acid precursors by desaturation, β–oxidation, reduction and oxidation.

**Results:**

We analyzed the composition of fatty acyl pheromone precursors and correlated it to the pheromone composition. Next, we investigated whether the downstream fatty–acyl reduction step modulates the ratio of alcohol intermediates before the final oxidation step. By isolating and functionally characterizing the Fatty Acyl Reductase (pgFAR) from each species we found that the pgFARs were active on a broad set of C8 to C16 fatty acyl substrates including the key pheromone precursors, *Z*9–14, *Z*9–16 and *Z*11–16:acyls. When presenting the three precursors in equal ratios to yeast cultures expressing any of the four pgFARs, all reduced (*Z*)–9–tetradecenoate preferentially over (*Z*)–11–hexadecenoate, and the latter over (*Z*)–9–hexadecenoate. Finally, when manipulating the precursor ratios *in vitro*, we found that the pgFARs display small differences in the biochemical activity on various substrates.

**Conclusions:**

We conclude that a pgFAR with broad specificity is involved in heliothine moth pheromone biosynthesis, functioning as a semi–selective funnel that produces species–specific alcohol product ratios depending on the fatty–acyl precursor ratio in the pheromone gland. This study further supports the key role of these in pheromone biosynthesis and emphasizes the interplay between the pheromone fatty acyl precursors and the Lepidoptera specific pgFARs in shaping the pheromone composition.

## Introduction

Many life forms depend on pheromones when finding a mate for reproduction [1],[2] but the detailed picture of the interplay between the genes, the enzymes and the pheromone production is barely understood. The chemical ecology of moths (Lepidoptera) has been thoroughly studied ever since the identification of bombykol, the sex–pheromone of *Bombyx mori* [3]. Most moths make use of long–chain fatty acid derivatives such as alcohols, acetates and aldehydes, and the vast majority of moths use a blend of pheromone components in specific ratios [4],[5]. Moth sex pheromones are produced in the female pheromone gland by a set of enzymes including β–oxidases, desaturases, fatty acyl reductases (FAR), oxidases, and acetyl transferases. Stereospecific members of the desaturase gene family have been extensively studied through gene characterization and expression analysis [6]–[Bibr pone.0037230-Roelofs1] [Bibr pone.0037230-Moto1] [Bibr pone.0037230-Serra1] [Bibr pone.0037230-Linard1] [11]. Recently, a few members of the reductase gene family have been discovered and functional assays have been developed to assess their biochemical activities [12]–[Bibr pone.0037230-Antony1] [Bibr pone.0037230-Lassance1] [15].

The four heliothine species *Heliothis virescens*, *Heliothis subflexa*, *Helicoverpa armigera*, and *Helicoverpa assulta* belong to a phylogenetic group of moths known as a major–pest lineage. *H. virescens* and *H. subflexa* are closely related species occurring in North and South America, while *H. armigera* and *H. assulta* are both occurring in Eurasia [16]. Like most heliothines, *H. virescens* uses a binary pheromone mixture composed of (*Z*)–11–hexadecenal (*Z*11–16:Al) as the major pheromone component, in combination with (*Z*)–9–tetradecenal (*Z*9–14:Al) as a minor compound, along with trace amounts of other aldehydes [17],[18]. Its closely related species, *H. subflexa* uses *Z*11–16:Al as major component in its pheromone and *Z*9–16:Al as a minor component along with (*Z*)–11–hexadecanol (Z11–16:OH), and also trace amounts of other aldehydes [19],[20]. *H. armigera* also uses *Z*11–16:Al as a major and *Z*9–16:Al as a minor pheromone compound, but with the latter in relatively smaller proportions to *Z*11–16:Al than was found in the pheromone of *H. subflexa*. Finally, *H. assulta*, the closely related species of *H. armigera*, differs in having *Z*9–16:Al as the major compound and *Z*11–16:Al as a minor component [21].

Similarly to many other moth species, pheromone biosynthesis in *H. virescens* starts from palmitic acid (16:Acid) that is converted to *Z*11–16:Acid by a Δ11–desaturase, which serves as substrate pool for both a FAR and a β–oxidase, producing *Z*11–16:OH and *Z*9–14:Acid, respectively [22],[23]. The latter is also further reduced to *Z*9–14:OH and both alcohols are oxidized to the corresponding aldehydes, *Z*9–14:Al and *Z*11–16:Al. *H. subflexa*, *H. armigera* and *H. assulta* also use this pathway toward *Z*11–16:Al. In *H. subflexa*, there seems to be no chain–shortening to produce *Z*9–14:Al. Instead, *H. subflexa* also produces *Z*9–16:Al by Δ11 desaturation of stearic acid (18:Acid) followed by chain–shortening, reduction, and oxidation [22]. In contrast, *H. assulta* mainly makes *Z*9–16:Acid from 16:Acid via a Δ9–desaturase, even though the pathway from 18:Acid is also active [21]. For all four species, a FAR is postulated to reduce the fatty–acyl precursors into their corresponding alcohols before oxidation to the aldehyde pheromone components. Thus, the four heliothine species use identical or biosynthetically related components and since the final pheromone blend ratios of the four species differ it is envisioned that either the FARs or the oxidases are involved in shaping these.

No moth alcohol oxidases active in pheromone biosynthesis have been characterized yet. On the other hand, the first pheromone gland specific FAR (pgFAR) was isolated from the silkmoth, *B. mori*, a 460–aa enzyme that is able to reduce Δ10,12–palmitoyl–CoA to the pheromone (*E,Z*)–10,12–hexadecadien–1–ol. This FAR is active on a broad range of saturated and monounsaturated C14– to C18–acyl precursors as well [12]. Antony et al. characterized a pgFAR from *Ostrinia scapulalis* that reduced the pheromone precursor (*Z*)–11–tetradecenoic acid to its corresponding alcohol [13]. Liénard et al. discovered that in three sister species of *Yponomeuta* a single pgFAR reduced a broad range of saturated and unsaturated C14– and C16–acyl precursors including the pheromone precursors, and with a preference for C14–substrates [15]. The chain length preference of the reductase together with the activity of an upstream Δ11–desaturase modulates the final ratio between the Δ11–unsaturated pheromone components [24]. The pgFARs' broad specificity in the Yponomeutidae and *B. mori* contrasts with findings in *Ostrinia nubilalis*, where two pgFAR alleles exist that encode two enzymes with a striking difference in stereoselectivity and which were proven to account for the difference between the two different pheromone races that have either (*E*)– or (*Z*)–11–tetradecenyl acetate as their major component and the other isomer as the minor component [14]. Similarly to the Δ11–desaturase subfamily [10],[25], the moth pgFAR orthologs are not found in any other organism and likely belong to a Lepidoptera–specific group of enzymes [14],[15].

The last biosynthetic step in the production of heliothine pheromones is the conversion of the fatty alcohols into their corresponding aldehydes by an alcohol oxidase. Teal and Tumlinson found that when topically applying various alcohols to the gland of *H. virescens* there was no specificity of the alcohol oxidase for saturated and unsaturated C14–16 precursors [26]. Wang et al. drew a similar conclusion when performing experiments with *H. armigera* and *H. assulta* [27]. These studies suggested that the alcohol oxidation involved in pheromone biosynthesis is not selective and thus that the modulation of the species–specific ratios must occur at an earlier point in the biosynthesis. In the present study, we investigated whether the final heliothine pheromone ratios derive from a single biosynthetic step or a combination of semi–selective steps and whether the reduction stage involves one or several active pgFARs. We report on the isolation of a *pgFAR* ortholog from each of the studied heliothine species and the activity of the encoded enzymes. We further tested whether the pgFARs have differences in their substrate preferences that may account for the final differences in blend composition or if they display a similar activity, which, depending on the species–specific precursor ratios, would mold the intermediate alcohol profiles, and thus the species–specific pheromone blends. We found that a single pgFAR is active in pheromone biosynthesis in each of the four Heliothines, and that these enzymes have a general selectivity for fatty acyl precursors within a range of C8–16, although with differences in substrate activity. Thus, the pgFARs are involved in molding the pheromone blends. In addition, our findings of pgFARs acting on a large variety of saturated and unsaturated fatty–acyl precursors support the idea that the functional flexibility of the pgFARs provides greater possibilities for evolving signal diversity [24].

## Methods

### 2.1. Insects

Larvae of the four heliothine moth species, *H. virescens*, *H. subflexa* and *H. armigera* (Bayer strain) and *H. assulta* originated from laboratory cultures maintained at the Department of Entomology, Max Planck Institute for Chemical Ecology, Jena, Germany and were fed on pinto bean, or soy bean artificial diets [28]. Male and female pupae were sexed and kept separately in a rearing chamber at 22±1°C under a 17–h:7–h light:dark photoperiod. Virgin females were separated daily before the scotophase and considered to be 0–day old.

### 2.2. Chemicals

(*Z*)–9–tetradecenoic methyl ester (*Z*9–14:ME) and (*Z*)–9–hexadecenoic methyl ester (*Z*9–16:ME) were purchased from Larodan Fine Chemicals AB (Limhamn, Sweden). The synthesis of (*Z*)–11–hexadecenoic methyl ester (*Z*11–16:ME) was previously described [14]. All FAMEs were dissolved in 96% ethanol in a 0.02 M stock solution. All alcohols used as reference compounds originated from our collection at Lund University.

### 2.3. Sex pheromone gland extracts and fatty–acyl precursor analyses

In virgin *H. virescens* females, the pheromone precursor content has been shown to peak at mid–scotophase in 2–days old individuals [29]. We examined the fatty–acyl lipid content in the four heliothine species. Pheromone glands (PGs) of 2– to 3–days old virgin females were dissected at mid–scotophase. The gland of a female was exposed by applying gentle pressure on its last abdominal segments and removed with sharp forceps or microscissors. Each gland was extracted for 30 min at room temperature (RT) in a glass vial containing 20 µl heptane and 0.5 ng/µl of pentadecyl acetate (15:OAc) as internal standard. The individual PG extracts were stored at −20°C until GC–MS analysis. The gland was transferred into a conical glass vial and its lipid content was extracted in 25 µl chloroform∶methanol (2∶1, v∶v) spiked with 250 ng of triheptadecenoin as internal standard. The reaction was incubated at 4°C overnight, then placed at room temperature and incubated for 1 h. The gland was removed and the extract was concentrated to dryness under a gentle nitrogen stream. The lipid extract was subjected to base methanolysis by addition of 25 µl of KOH (0.5 M in methanol) and mixed thoroughly, then incubated for 3 h at RT. The reaction was acidified by adding 25 µl HCl (1 M in water) followed by addition of 25 µl hexane. The samples were shaken, left to separate for 2 min, and then the hexane phase was transferred to a clean glass insert contained in a 2 ml–glass vial and stored at 20°C prior to GC–MS analysis.

### 2.4. Gas chromatography and mass spectrometry (GC–MS) analyses

A Hewlett Packard HP 5890II GC system, coupled to a mass selective detector (HP 5972) and equipped with a medium-polar INNOWax column (100% polyethylene glycol, 30 m×0.25 mm I.D., film thickness 0.25 µm, Agilent Technologies) was used. The GC–MS was operated in electron impact mode (70 eV), the injector was configured in splitless mode at 220°C, and helium was used as carrier gas (velocity: 30 cm/s). The oven temperature was maintained for 2 min at 50°C and increased at a rate of 10°C/min up to 220°C, and held for 20 min. The fatty alcohols, aldehydes, and fatty acid methyl esters (FAMEs) were identified by their retention time, their mass spectra and by comparison with reference compounds. The relative ratio (%) of the three pheromone methyl esters *Z*9–14:ME, *Z*9–16:ME, and *Z*11–16:ME, as well as the ratio of their corresponding alcohols in the gland extracts were calculated based on manual integration of the chromatogram peak areas using the Enhanced ChemStation® software (Agilent Technologies).

### 2.5. RNA extraction, cDNA synthesis, and amplification of the full–length FAR sequences

Glands were excised from 1 to 4 day–old females from each of the four species and total RNA extracted according to the instructions given by the manufacturer (RNeasy mini kit, Qiagen). First strand PG cDNAs were synthesized from 1 µg of total RNA with a reverse transcriptase (Stratascript, Stratagene, AH Diagnostics, Skärholmen, Sweden) and were used as template in subsequent PCR reactions. The *Heliothis virescens FAR* (*HvFAR*) nt sequence [30] was downloaded from GenBank (accession nr. EZ407233), and by performing a BLAST search in *H. armigera* (Har) genomic DNA local 454 database (Max Planck Institute for Chemical Ecology, Jena, Germany) we found a hit (e value 7e^−52^) to a partial FAR orthologous sequence, HarFAR contig90541. Two gene–specific primers (GSPs), pFlHvFARs and pFlHvFARas (Table S1) were designed in BioEdit [31] from the full–length *HvFAR* sequence information in order to amplify the corresponding ORF. The *HvFAR* ORF was amplified from 100 ng of PG cDNA in a 20 µl PCR reaction (containing 3.5 mM MgCl_2_, 0.5 µM of each GSP primer, 0.4 ul Advantage 2 polymerase mix and 0.4 µl dNTPs 10 mM), with the following cycling program: 95°C for 5 min (1×), denaturation at 95°C for 30 s, annealing at 60°C for 30 s, and elongation at 72°C for 3 min (35×), then an additional elongation step at 72°C for 10 min. The PCR products were analyzed on a 1% TAE agarose gel with a GeneRuler 100–bp plus ladder (Fermentas, Helsingborg, Sweden). The GSPs designed from *HvFAR* successfully amplified the orthologous *pgFAR* sequence from *H. subflexa* (Hs) and *H. armigera* (Har) using Taq polymerase (Metabion) under similar PCR conditions. For *H. assulta* (Has), the combination of pFlHvFARs and HvFAR1078_710R and Has PG cDNA amplified a DNA fragment of ca 1 kb. To obtain the remaining 3′ sequence we designed degenerate primers (HFAR3URTI–III) from the consensus 3′UTR sequences from *HvFAR* and HarFAR contig90541. The PCR products were cleaned with the two enzymes Exo and SAP and sequenced in both directions with the BigDye® v3.1 cycle sequencing kit. To ensure that the different Hv primers did not introduce a bias in the 3′ and 5′ ends of the *Hs*, *Har*, and *HasFAR* nucleotide sequences, we designed internal GSPs based on each species' orthologous pgFAR sequence to confirm the entire ORF sequence. Using various primer combinations (Table S1) and 3′ and 5′ RACE ready cDNAs synthesized according to instructions provided in the SMART RACE cDNA Amplification Kit (Clontech), we amplified the 3′ and 5′ ends of all *pgFARs* and confirmed their ORF nt sequences.

### 2.6. Sequence analyses and gene tree construction

DNA sequence analyses and comparisons of amino acid sequence similarities were performed using BioEdit Sequence Alignment Editor software v.7.0.5.3 [31]. The DNA sequences were compared to nucleotide collections and public non–redundant databases of Blastn, Blastx, and Blastp [32]. Multiple sequence alignments of deduced amino acid sequences were performed with the ClustalW2 algorithm [33] followed by manual inspection. The building of the Neighbor–joining gene tree was done with MEGA v.4.0.2 (JTT model, 1500 replicates, pairwise comparisons) [34] on the deduced aa sequences of FARs from various arthropods and animals retrieved from the NCBI Protein Database or as stated in Liénard et al. [15].

### 2.7. Functional single substrate assay in yeast

Each full–length FAR ORF was amplified using gene–specific primers (Table S1) and cloned in the pYES2.1 expression vector downstream of the GAL1 promoter according to the instructions given by the manufacturer (Invitrogen) before confirmation by sequencing with the vector specific primers Gal1 and V5. The four pgFAR constructs and the sole pYES2.1 plasmid were transformed into the *InvSc1* strain of *S. cerevisiae* (Invitrogen) and grown on SC–U plates with 0.7% YNB (w/o aa, with ammonium sulphate), and a drop–out medium lacking uracil (ForMedium™ LTD, Norwich, England), and 2% glucose. Single autotrophic colonies were inoculated in 5 mL SC–U medium and incubated for 24 h at 30°C and 300 rpm (Innova 42, New Brunswick Scientific), then diluted to an OD_600_ = 0.4 to a final volume of 20 mL in SC–U medium containing 2% galactose and 0.1% glucose in 250–mL flasks, and incubated for 24 h at 30°C and 300 rpm. Then 0.5 mM alcohol–free ME precursors were added to the yeast cultures diluted to 1∶10 in 2 mL SC–U 2% galactose, 1% tergitol (Nonidet P–40, Sigma) following by incubation for 24 h at 30°C and 300 rpm. Mixtures of alcohol products are found both in the yeast cell pellets and the culture medium in identical proportions [15] and similarly to previous studies, we extracted alcohols from the yeast pellet only [12],[14],[15]. Briefly, cells were collected by centrifugation at 2,000× *g* (Labofuge 200, Heraeus Instruments), and the cell pellets were extracted with 1 mL *n*–hexane including 150 ng Z11–13:OH as an internal standard followed by shaking at 200 cycles/min (Vibramax 100, Heidolph) for 1 h. The hexane layer was recovered and samples were stored at −20°C until gas chromatography analyses, prior to which they were concentrated under a gentle flow of N_2_ to around 50 µl.

### 2.8. FAR multiple substrate ratio assay

The FAR yeast transformants were inoculated and grown as previously mentioned. A ratio of the three precursors *Z*9–14:ME, *Z*9–16:ME, and *Z*11–16:ME was added in a total concentration of 0.5 mM. Three different ratios (Ratio 1–3, Table 1) with three replicates for each ratio, plus a negative control (vector only) were initially tested. Ratio 1 was tested to assess if the different pgFARs have an identical substrate preference on a mixture of compounds supplied in equal proportions as to when the compounds are supplemented individually. We then used two different precursor ratios to test if each orthologous pgFAR was able to modulate the alcohol production (Ratio 2–3). A final ratio assay based on the pheromone gland precursor analysis was performed (Ratio 4, Table 1). Incubation, extraction and GC–MS analysis were performed as previously described. The three peaks on the chromatogram corresponding to *Z*9–14:OH, *Z*9–16:OH, and *Z*11–16:OH were manually integrated to calculate their relative ratio.

**Table 1:** The relative methyl ester (ME) ratios used in functional assays.

Ratio	*Z*9–14:ME	*Z*9–16:ME	*Z*11–16:ME
1	0.333	0.333	0.333
2	0.04	0.48	0.48
3	0.01	0.97	0.02
Hv–ratio 4	0.07	0.31	0.62
Hs–ratio 4	0	0.62	0.38
Har–ratio 4	0	0.49	0.51
Has–ratio 4	0	0.94	0.06

*The total concentration of ME in the assays was kept constant at 50 mM. Ratio 1–3 were used in assays for all four pgFARs, while ratio 4 was determined based on the gland precursor ratios found in the individual species.

### 2.9. Statistics

Calculations of Standard Deviations (SD) and Standard Errors of the Mean (SEM) were made in Microsoft Office Excel. All following statistical analyses were calculated with IBM SPSS v.19.0.0. Test for normal frequency distribution was made with Q–Q plot. The values tested were the relative proportions compared to the most abundant compound (set to 1) and significant differences between the ME and alcohol precursors and their corresponding aldehydes in the glands, and for the ratio assays MEs and the alcohol products, were determined by means of a one–way Analysis of Variance, including Tukey's test and Homogeneity of Variance Test. If there were unequal variances of the sample sets, we performed the Welch and Brown–Forsythe tests of Equality of Means, and a Games Howell-test as an alternative to Tukey's test.

## Results

### 3.1. Pheromone gland extracts and fatty acid precursor analyses

**Figure 1:**
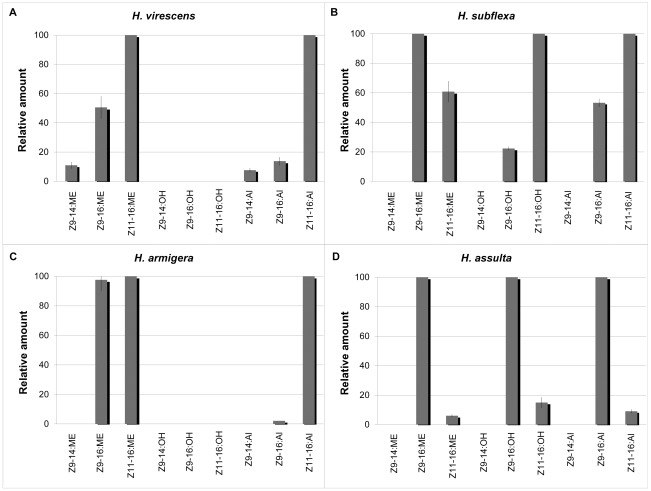
Gland analysis of the pheromones and pheromone precursors in the four heliothines. Relative amounts of *H. virescens* (A), *H. subflexa* (B), *H. armigera* (C), and *H. assulta* (D) female pheromone gland compounds, *i.e.*, the *Z*9–14, *Z*9–16, and *Z*11–16–acid precursors and their corresponding alcohol and aldehyde forms, the latter representing the active pheromone components. The aldehyde ratios of *H. armigera* are based on values from Wang et al. [21]. Lines represent the standard error of the means.

In all glands of the four heliothine species *H. virescens*, *H. subflexa*, *H. armigera*, and *H. assulta*, we found the common FA derivatives such as palmitic acid, stearic acid, oleic acid, linoleic acid, and linolenic acid. Besides these compounds, the different species displayed unique profiles of pheromone precursor ratios. The *H. virescens* pheromone gland contained three aldehydes, *Z*9–14:Al, *Z*9–16:Al and *Z*11–16:Al, in a 8∶14∶100 ratio on average (N_PG samples_ = 7). The first and latter aldehydes constitute the attractive pheromone blend [17],[18]. The saturated 16:Al and 14:Al were also found (data not shown), similarly to previous studies [18],[29],[35]. GC–MS analysis of a methanolysed gland lipid extract showed that the corresponding methyl ester precursors were found in a 11∶50∶100 ratio (Figure 1A). To test whether the pheromone and precursor ratios differ significantly in this species, we performed the Welch and Brown–Forsythe tests (due to unequal variances), in which we compared the proportions of either *Z*9–14 or *Z*9–16 aldehydes to their corresponding fatty acyl precursors. A difference between fatty-acyl precursor and pheromone component ratios would suggest that downstream biosynthetic enzymes are influencing the relative proportions in the final blend. There was no statistical difference in the proportions between *Z*9–14:Al and *Z*9–14:Acid relative to the *Z*11–16–derivatives. However, the relative abundance of *Z*9–16:Al was significantly lower than its precursor (P = 0.002, F(1,12) = 20.997, P = 0.001).

The *H. subflexa* pheromone glands contained the two pheromone components *Z*9–16:Al and *Z*11–16:Al (N_PG samples_ = 11), in an average ratio of 53∶100 (Figure 1B). These are the attractive components in this species (together with Z11–16:OH) [19],[20]. The two fatty acyl pheromone precursors, *Z*9–16:ME and *Z*11–16:ME (N_PG samples_ = 12) were present in a ratio of 100∶61, while the corresponding fatty acyl alcohols (N_PG samples_ = 10) were found in a 22∶100 ratio. The N–values of the samples differ since the aldehydes and/or alcohols were below the limit of quantification in some of the glands. When comparing the relative proportions of *Z*9–16:Acid to its derived *Z*9–16:OH and *Z*9–16:Al the precursor appeared to be significantly (F(2,29) = 54.585, P = 0.001) more abundant than both the corresponding alcohol and aldehyde (P<0.001). The *Z*11–16:Acid was present in smaller amount compared to *Z*9–16:Acid, while both the corresponding alcohol and aldehyde showed the opposite pattern, being significantly more abundant than the *Z*9–16 compound (F(2,29) = 26.518, P<0.001). The *Z*9–16 alcohol and aldehyde did not differ in relative abundance (P>0.05).

The *H. armigera* glands contained the major pheromone component *Z*11–16:Al, with traces of *Z*9–16:Al, which is in agreement with the 100∶2 to 100∶7 ratio of pheromone components previously reported [21]. The precursors *Z*11–16:ME and *Z*9–16:ME (N_PG samples_ = 9) were present in a 100∶98 ratio (Figure 1C). When comparing the relative proportion of *Z*9-16:Acid to its corresponding aldehyde, the former was significantly more abundant than the latter (F(1,16 = 113.98, P<0.001). There was no significant difference between the relative proportion of *Z*11–16:Acid and *Z*11–16:Al (P>0.05).

In *H. assulta*, the glands contained the major pheromone component *Z*9–16:Al and the minor *Z*11–16:Al (N_PG samples_ = 7) in a ratio of 100∶9, which agrees with previously reported ratios [21]. The corresponding alcohols were not found in all gland extractions, but in those that did have them (N_PG samples_ = 4) they were present in a 100∶15 ratio, and the ME precursors (N_PG samples_ = 5) in a 100∶6 ratio (Figure 1D). There was a significant difference between the ratios of the *Z*11–16–derivates in relation to the *Z*9–16 derivates (F(2,13) = 4.708, P = 0.029), with *Z*11–16:OH being present in significantly larger amounts than its the corresponding acid (P = 0.024).

### 3.2. Cloning of pheromone gland biosynthetic fatty–acyl–CoA reductases (pgFARs)

**Figure 2:**
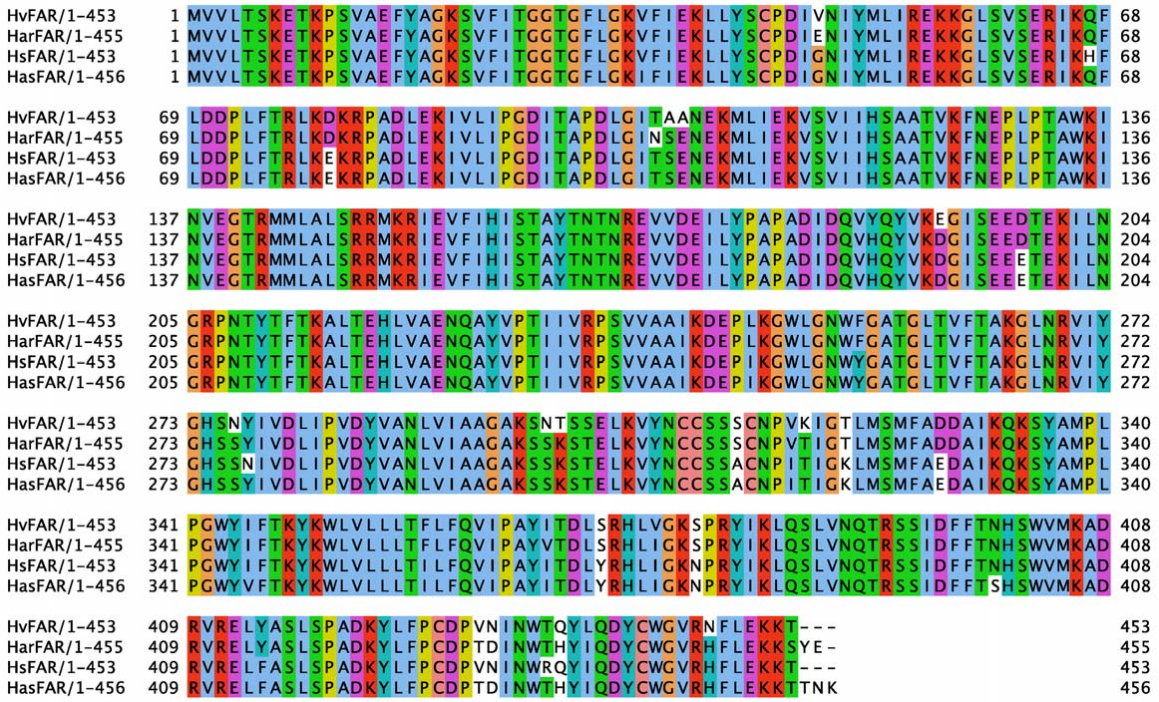
Multiple alignment of HvFAR, HsFAR, HarFAR, and HasFAR. Multiple alignment of the four FAR aa sequences from *H. virescens*, *H. subflexa*, *H. armigera*, and *H. assulta*. Clustal color code indicates conserved aa positions and white background reflect non–conservative aa substitutions.

**Figure 3:**
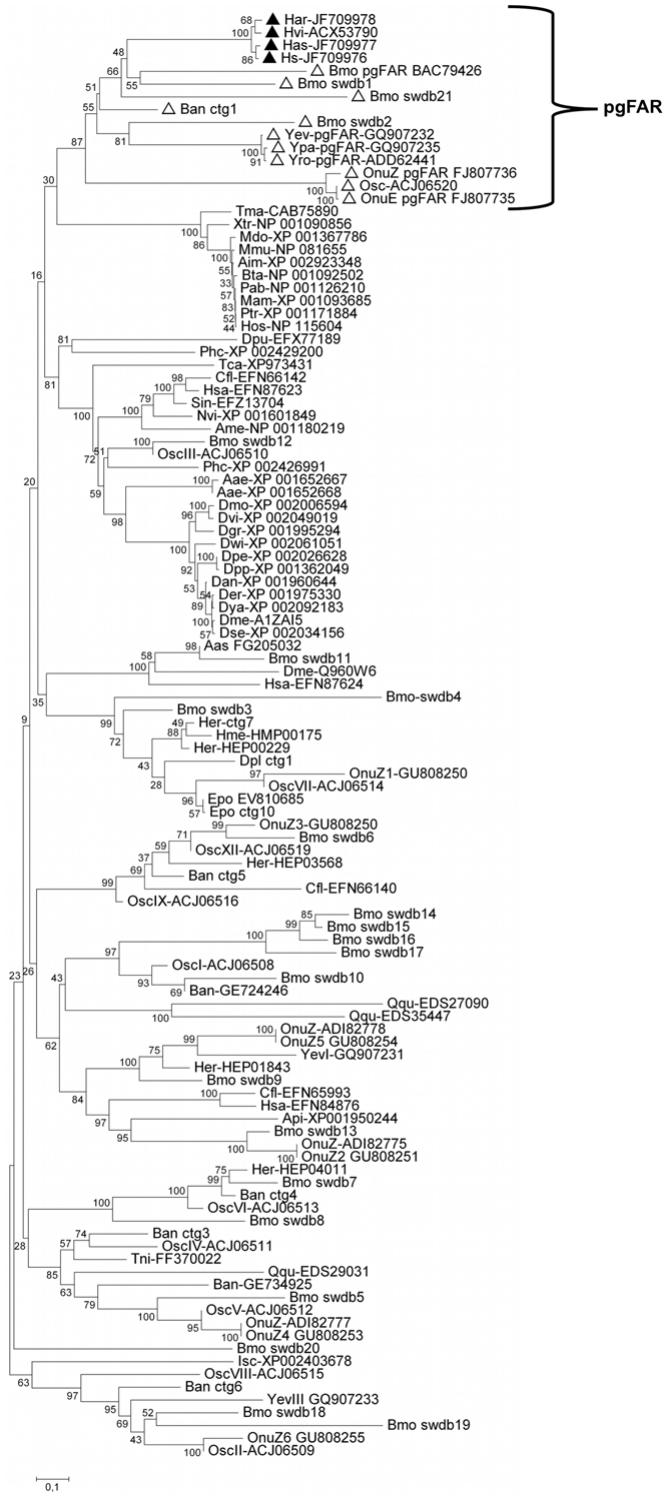
FAR gene tree. Gene tree of arthropod, mammalian and lepidopteran FARs including the lepidopteran–specific pgFAR group, supported with a bootstrap value of 87 and marked with a bracket. Within this group, heliothine pgFARs are marked with dark triangles and other biosynthetic moth reductases with transparent triangles. The Neighbor–joining algorithm analysis was computed in MEGA (v. 4.0) using deduced aa sequences with pairwise deletion, and the JTT matrix based model with 1,500 bootstrap replicates. Sequences were retrieved from GenBank by manually searching for arthropod FARs, as well as BLASTP database searches using HvFAR as query. *B. mori* FAR sequences were retrieved from the Silkworm Genome Database. All sequences noted as cng (contig) were obtained from Liénard et al. [15]. The full species names can be found in (Table S2). Sequences were aligned with the ClustalW2 algorithm with the ClustalX2 interface and manually inspected before computing the phylogenetic relationship.

The *H. virescens* and *H. subflexa* FARs, HvFAR and HsFAR (accession no. JF709976) respectively, displayed a 1362 nt open–reading frame (ORF) that translated into a 453 aa–protein, while the *H. armigera* and *H. assulta* homolog FARs, HarFAR and HasFAR (accession no. JF709978 and JF709977), encompassed an ORF of 1367 nt and 1374 nt, which corresponded to 456 and 457 aa–proteins, respectively. Comparisons between aligned sequences at the nt and the aa level revealed a high level of sequence identity, ranging from 86.3 to 91.9, and 92.5 to 97.4% respectively as shown in Table 2 and Figure 2. All the deduced protein sequences contained the Rossmann–fold NAD(P)H–binding protein domain and the C–terminal domain of fatty–acyl–CoA reductases, as confirmed by BLASTP searches and PFAM protein domain search [36]. A gene tree was constructed using a set of FAR sequences from a wide range of non–insect and insect organisms. All newly isolated heliothine FAR candidates clustered within the lepidopteran pgFAR clade that contains FARs involved in sex pheromone biosynthesis (Figure 3).

**Table 2:** Identity between the pgFAR nucleotide and amino acid sequences.

*Nt*	HvFAR	HsFAR	HarFAR
HsFAR	93.8		
HarFAR	95.4	94.7	
HasFAR	92.5	97.4	95.2
*aa*			
HsFAR	88.5		
HarFAR	91.9	88.3	
HasFAR	86.3	91	89.5

### 3.3. Heterologous expression in yeast

**Figure 4:**
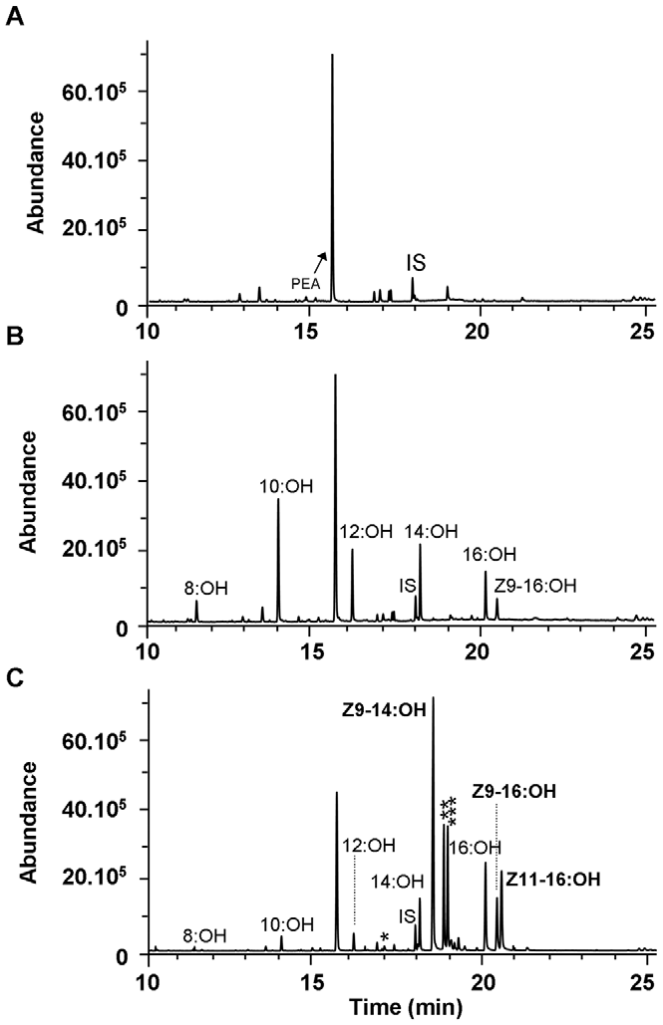
Functional assay and GC–MS analysis of HvFAR. Typical total ion current (TIC) chromatograms from yeast cells expressing the pYES2.1 control (A), the *H. virescens* pgFAR (HvFAR) (B) and HvFAR in the functional assay with a blend of the three biosynthetic precursors in equal concentrations (ratio 1) (C). The control yeast produces no fatty alcohols (A) whereas the yeast expressing HvFAR convert a series of fatty acyls into their corresponding fatty alcohols (B–C). PEA refers to phenylethyl alcohol, a natural yeast aroma compound present in the extracts. The asterisks in (C) indicate the remaining methyl ester precursors (*, *Z*9–14:ME; **, *Z*9–16:ME and *** *Z*11–16:ME). The internal standard (IS) corresponds to 150 ng of *Z*11–13:OH.

No alcohol products were found in yeast extracts prepared from the negative control (Figure 4A). In contrast, yeast samples expressing either FAR contained various amounts of alcohols corresponding to saturated 8:Acid, 10:Acid, 12:Acid, 14:Acid, and 16:Acid occurring naturally in the yeast (Figure 4B). The heliothine pgFARs were not able to reduce substrates longer than C16 in chain length. In addition, we also found minor amounts of *Z*9–16:OH in all samples, indicating that all four enzymes were able to reduce the *Z*9–16:Acid produced by the yeast (Figure 4B). When adding either *Z*9–14:ME or *Z*11–16:ME at a same concentration (0.5 mM) to yeast cultures expressing either of the four pgFAR candidates, GC–MS analyses of yeast extracts showed that the encoded FAR enzymes were able to reduce the acyl substrates into their corresponding alcohols, namely the *Z*9–14:OH and *Z*11–16:OH. The relative amounts of *Z*9–14:OH were higher compared to *Z*11–16:OH for all FAR constructs tested, which indicates an overall substrate preference for the *Z*9–14:ME. When supplementing the yeast expressing either FAR with the three unsaturated biosynthetic acyl precursors together, each enzyme accordingly reduced them to their corresponding alcohols (Figure 4C) and converted proportionally less of the saturated yeast acyls. Altogether this demonstrated that the heliothine FAR candidates encode active pheromone biosynthetic pgFARs with a broad activity on various saturated and unsaturated substrates ranging from C8 to C16 in chain length.

### 3.4. Ratio assays

**Figure 5:**
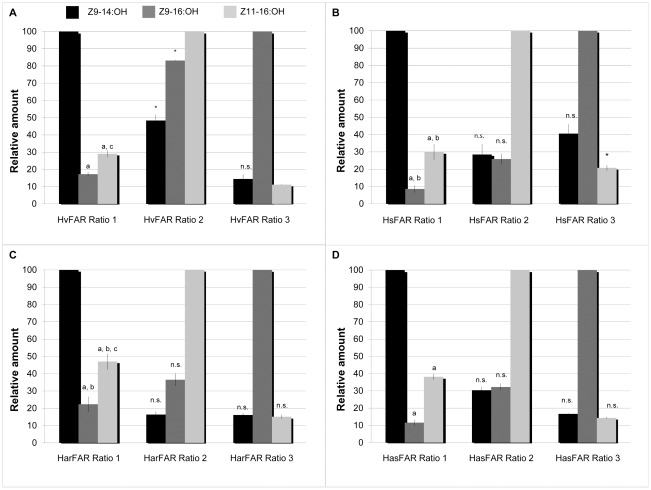
Multi-substrate assays of the four heliothine pgFARs. The graphs represent (A) the produced amounts of *Z*9–14:OH, *Z*9–16:OH, and *Z*11–16:OH, illustrated as relative amounts from the functional assays of HvFAR, (B) HsFAR, (C), HarFAR and HasFAR (D). Bars represent the standard error of the mean. These results show that the heliothine pgFARs are broad range enzymes with a substrate preference for Z9–14:acyls followed by Z11–16 and then Z9–16 acyls, and that there is a trend for all pgFARs that the most abundant precursor will be converted to the major product in vitro. The used precursor ratios are referred to in Table 1. The marking a equals P<5% to Z9–14:OH, b P<5% between HsFAR–HarFAR, c P<5% HvFAR–HarFAR, and * equals a significant difference of the marked bar to all other FARs. Non–significant results are marked with n.s.

The alcohol profiles obtained from assays performed with variable amounts of *Z*9-14, *Z*9-16 and *Z*11-16:ME are shown in Figure 5 A–D. When supplementing a mixture of the three precursors in identical proportions (Ratio 1: 0.333∶0.333∶0.333 for *Z*9–14:*Z*9–16:*Z*11–16), all pgFARs produced significantly more *Z*9–14:OH than *Z*9–16:OH (F(3,8) = 5.255, P = 0.027) or *Z*11–16:OH (F(3,8) = 5.661, P = 0.022), and significantly more *Z*11–16:OH than *Z*9–16:OH (F(1,22) = 40.889, P<0.001) (Figures 4 and 5 A–D). This indicates that all pgFARs display a common overall substrate preference for the C14 acyl substrate over the two biosynthetic C16 homologs. In addition, it can be noted that HarFAR produced significantly more *Z*9–16:OH than HsFAR (Tukey, P = 0.027), and more *Z*11–16:OH than HvFAR and HsFAR did (Tukey, P = 0.029 (Hv–Har), P = 0.036 (Hs–Har)). The overall activity of HarFAR consequently seemed to be enhanced compared to HvFAR and HsFAR. The HasFAR alcohol production was intermediate and it was not significantly different from either of the extremes (P>0.05).

When decreasing the relative proportion of *Z*9–14:ME in the supplied mixture (Ratio 2, 0.04∶0.48∶0.48), all pgFARs produced *Z*11–16:OH as the major alcohol product (Figure 5 A–D). This supports our observations from the assay with ratio 1 that all FARs preferentially reduce the *Z*11–16 over its *Z*9–16:acyl homolog. Interestingly, the FARs exhibited a difference in their overall production of both *Z*9–14:OH and *Z*9–16:OH compared to *Z*11–16:OH: HvFAR produced higher amounts of *Z*9–14:OH (F(3,8) = 12.313, P = 0.002) and *Z*9–16:OH (F(3,8) = 103.398, P<0.001) compared to the other three FARs (*Z*9–14:OH: P = 0.024 (Hs–Hv), P = 0.001 (Har–Hv), and P = 0.039 (Has–Hv); *Z*9–16:OH: P<0.001 for all).

When dramatically increasing the relative proportion of the least preferred substrate (*i.e.* the *Z*9–16:ME) in the three–component mixture (Ratio 3, 0.01∶0.97∶0.02), the alcohol profile accordingly shifted towards high proportions of *Z*9–16:OH (Figure 5 A–D). Altogether, experiments with Ratio 1–3 suggest that the heliothine pgFARs can affect the alcohol outcome in combination with variable proportions of the biosynthetic precursors, despite having a broad activity on various substrates.

**Figure 6:**
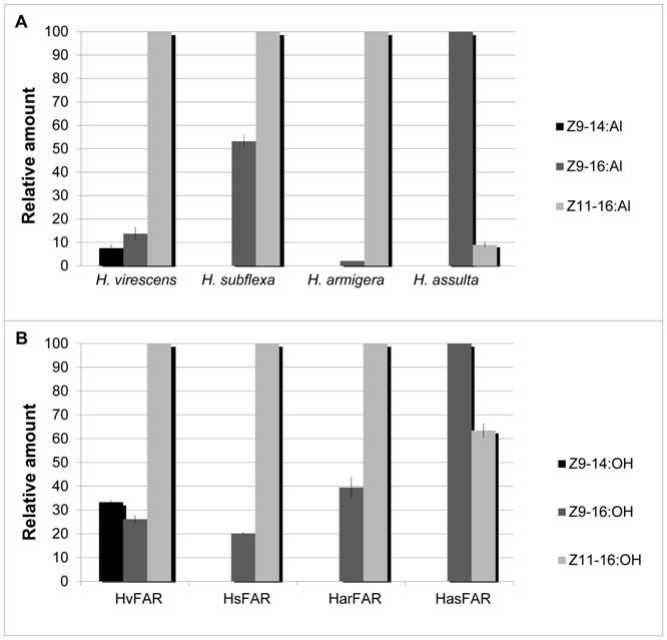
Modulation of the alcohol profile in a ratio-assay matching gland precursor amounts. A. Pheromone gland: The relative percentage ratio of the pheromone content of *H. virescens*, *H. subflexa*, *H. armigera*, and *H. assulta* for *Z*9–14:Al (*H. virescens* only), *Z*9–16:Al, and *Z*11–16:Al. The standard error of the mean is shown as a line in the bars. B. Yeast cells: The relative percentage ratio of *Z*9–14:OH (present in HvFAR only), *Z*9–16:OH, and *Z*11–16:OH produced when the pgFARs are expressed heterologously in yeast with addition of the corresponding precursors in ratios derived from pheromone gland analysis. The precursor ratios can be found in Table 1 as Ratio 4 for each species.

In a fourth ratio assay, we supplemented the yeast with the methyl ester precursors in proportions matching those of the biosynthetic precursors in each species' female gland (see Figure 1, Table 1). The relative amounts of alcohols produced are shown in Figure 6. When supplementing yeast expressing the HvFAR with the Hv–ratio 4 (0.07∶0.31∶0.62), the resulting relative amounts of *Z*9–14:OH, *Z*9–16:OH, and *Z*11–16:OH were 33.3∶26.2∶100, while the *H. virescens* female gland contained the three corresponding aldehydes in a 7.6∶13.8∶100 ratio. When supplementing yeast expressing the HsFAR with the Hs–ratio 4 (0∶0.62∶0.38), yeast samples contained a final alcohol mixture of Z9–16:OH and Z11–16:OH in a 20.2∶100 ratio, which matched the alcohol ratio found in *H. subflexa* female glands, but did not fully match the corresponding aldehydes, which are found in a 53.2∶100 ratio. When applying the Har–ratio 4 (0∶0.49∶0.51) to yeast expressing the HarFAR, the enzyme produced *Z*9–16:OH and *Z*11–16:OH in a 39.5∶100 ratio, as compared to the reported 2.1∶100 aldehyde ratio [21]. Likewise, when assaying yeast bearing the HasFAR construct with the Has–ratio 4 (0∶0.94∶0.06), composed of a mixture of *Z*9–16 and *Z*11–16:acyl precursors, the corresponding alcohols were found in a 100∶63.4 ratio, compared to the aldehyde ratio of 100∶9. Altogether, our results from the assays confirm that the heliothine pgFARs have a substrate preference for *Z*9–14>*Z*11–16>*Z*9–16 acyl precursors, and that they produce a different ratio of alcohol products depending on the supplied precursor ratio. However, the alcohol ratios measured *in vitro* did not entirely match the ratios of aldehyde pheromone components *in vivo* in the respective heliothine species.

## Discussion

The Heliothine pgFARs are broad-acting, semi-selective enzymes involved in molding the pheromone composition. In moth pheromone glands, the fatty acid pool is derived from the combined action of desaturases and β–oxidases. The fatty–acyl moieties subsequently undergo reduction [4],[5],[23],[37]. To determine if the reduction step influences the composition of the specific pheromone blends in *H. virescens*, *H. subflexa*, *H. armigera*, and *H. assulta*, we identified and functionally characterized the pgFAR of each species. These key biosynthetic enzymes convert fatty–acyl precursors into their corresponding primary alcohols [38], and are involved in shaping pheromone ratios in other moth species [14],[15]. In all four heliothines we found that the ratios of the final compounds differ compared to the fatty acyl precursor ratios, most notably in *H. armigera* and *H. subflexa*. *In vitro*, the four FARs are able to reduce a broad range of C8–16 substrates. In addition to this broad specificity, the four pgFARs are selective with respect to chain length and double bond position: when the enzymes were tested on a blend of compounds, the largest amount produced was that of *Z*9–14:OH, followed by *Z*11–16:OH and *Z*9–16:OH for all four pgFARs. When varying the precursor ratios, we observed that the amount of an alcohol product depended on the supplied proportion of its precursor in relation to the other precursors. Hence, the alcohol product ratio resulting from the action of each pgFAR will differ depending on the amount of precursors present in the respective species' female gland, due to the hierarchical preference of the enzyme. This supports that the heliothine pgFARs to some extent molds the final proportions of components in the different species' pheromone blends.

The composition of pheromone compounds in the glands of *H. virescens, H. subflexa, H. armigera*, and *H. assulta* in our study agrees to what was previously published [17]–[Bibr pone.0037230-Klun1] [Bibr pone.0037230-Klun2] [Bibr pone.0037230-Teal2] [21]. Interestingly, our analyses of the corresponding fatty acid precursors revealed that in all species, but *H. assulta*, the final pheromone blend ratios differed from the precursor ratios. This indicates that the reduction and/or oxidation step modulates the pheromone composition. However, a modulating effect at the oxidation step is unlikely, as the oxidase was found to be unspecific [26],[27], whereas pgFARs in other species, both highly specific and general specific reductase enzymes, have been shown to modulate the intermediate fatty alcohol profiles both *in vivo* [39] and *in vitro* [14],[15].

The full–length FAR ORF sequences from each species were highly conserved both at the nt and aa levels (Figure 2). These four orthologous FAR candidates clustered together with gene members of the sub–family of pheromone biosynthetic lepidopteran pgFARs from which no apparent ortholog FAR sequences are found in other arthropods and mammals. These findings indicate that moths have recruited and evolved a specific group of FARs for the sole purpose of pheromone biosynthesis, and that a single FAR is active in heliothines, similarly to findings from *Bombyx*, *Ostrinia* and *Yponomeuta* [12]–[Bibr pone.0037230-Antony1] [Bibr pone.0037230-Lassance1] [15]. Interestingly, the heliothine pgFARs cluster in the gene tree in proximity to both the *B. mori* and *Yponomeuta* spp. pgFARs that are enzymes of broad specificity (Figure 3), while the *Ostrinia* spp. pgFAR orthologs are found in a separate subgroup of more selective or even specific reductases. Interestingly, neither the *Yponomeuta* spp. pgFAR nor the heliothine pgFARs are able to reduce any substrate larger than C16. If this is just an incidental consequence of the enzyme's structure (size of the binding pocket, positions of the catalytic residues, etc.) or if there is an adaptive explanation for these FARs not being able to act on shorter/longer chain–lengths remains unknown for now. By outgroup comparison it however appears likely that the ancestral lepidopteran pgFAR was a general specific pgFAR. The use of a functionally flexible ancestral pgFAR active on several pheromone precursors may have facilitated the evolution of novel moth pheromones as long as new precursors are made available upstream in the biosynthetic process [24]. The subgroup of mammalian FARs clusters in proximity to the pgFARs, and although the bootstrap value is low it is interesting to note that the mammalian orthologs convert saturated and unsaturated C16-18 fatty acyl substrates [40].

In a single-substrate-assay, the activities of the various pgFARs were not strikingly different, and thus it is most likely an interplay of a defined precursor ratio and enzymes' substrate preference that produces the unique alcohol profiles prior to the oxidation step. Our results from the assay using the three major pheromone precursors in identical relative proportions/concentrations (Figure 5, ratio 3) reveals the pgFARs' significant substrate preference for the *Z*9–14:ME over both *Z*9–16:ME and *Z*11–16:ME.

Multi substrate assays can be used to measure enzyme/substrate specificity constants and are consistent with individual measurements [41]. As long as the assay conditions remain the same for all single experiments, multi substrate assays can be used to screen the model enzymes [42]. In addition, the main characteristics, such as biomass, dry weight, glucose flux, and mRNA levels of glycolytic enzymes, in yeast that is cultured under constant conditions, where are usually comparable [43]. This supports our ratio assay as a reliable technique for investigating activity patterns of moth biosynthetic pgFARs.

Interestingly, in our multi-substrate assays, we observed topical differences in HvFAR, HsFAR and HarFAR reductive activity, which we postulate arise when the substrates compete for the enzyme's binding pocket, as simulated in our assays. For instance, when presenting a precursor blend with the preferred substrate *Z*9–14:ME in a minor proportion compared to *Z*9–16:ME and *Z*11–16:ME (Figure 5, Ratio 2), the enzymes accordingly produce more *Z*11–16:OH, the second preferred substrate. But differences between enzymes were emphasized, i.e., HvFAR produced more *Z*9–14:OH and *Z*9–16:OH than the other three enzymes. With high amounts of *Z*9–16:ME (Figure 5, Ratio 1), all enzymes produce more Z9-16:OH but still individual differences occur as HsFAR produced more Z11–16:OH than the other pgFARs. This shows that differences in the biochemistry between the four pgFARs affect the alcohol production from multi–substrate precursor blends, and that the outcome of the reductive step in the pheromone biosynthesis of the four moths is dependent on a certain precursor ratio in a complex environment. It is known that the concentration of a substrate affects an enzyme's activity, and by extension, more substrates further adds to the complexity of the system [44],[45]. If we consider that the different pheromone precursors bind to the same active site of the FAR enzyme, they can be regarded as competing molecules. These will temporary prevent the enzyme from acting on the other substrates since the possible complexes of an enzyme, a substrate and competing molecules are “enzyme–substrate” or “enzyme–inhibitor”, but never “enzyme–substrate–inhibitor” [44]. For the heliothine pgFARs, the enzyme–substrate may either be “pgFAR–*Z*9–14:Acyl”, “pgFAR–*Z*9–16:Acyl”, or “pgFAR–*Z*11–16:Acyl”. Thus the complex formation varies depending on the ratio of substrates that consequently affects the resulting alcohol profiles (Figure 5), which may explain minor differences between the activity levels of the four pgFARs in some of the assays, but does not affect our main findings or the reliability of our data.

The cell environment may differ between a moth pheromone gland and the yeast, which can affect the enzyme activity *in vitro* versus *in vivo*. This is a possible explanation for the result in the experiment with ratio 4. Here the pgFARs are provisioned with the species–specific gland–derived fatty–acyl precursor ratio (Figure 5, Ratio 4), and the fatty–alcohol ratio produced *in vitro* became closer, albeit not identical to the final pheromone composition in each species. In addition, a yeast cell contains other competing substrates such as saturated or unsaturated C8–16 [46], which can be reduced by the pgFARs, as well as factors that may have an inhibitory effect on the enzyme's folding or activity [47]. Saturated aldehydes and alcohols were also present in the gland extracts, indicating that saturated acyls are also reduced in the insect. A certain proportion of the *Z*9–16:Acid in the pheromone gland samples may result from metabolic fatty acid production, and may be used only to some extent to pheromone biosynthesis [4], therefore potentially causing a relative bias in the calculated ratio among the three biosynthetic precursors. Finally, it remains unexplored to date how the precursors are transported to the pheromone enzymes in the gland and if non–pheromone precursors are subjected to the pgFARs.

A precise pheromone blend is however rarely the outcome of a single gene [14], and usually results from the combined activity of several biosynthetic enzymes including desaturases, reductases and/or oxidases or acetyl transferases [5],[24],[48]. Crossing experiments have shown that the difference between the pheromone blends in *H. assulta* and *H. armigera*, which use the *Z*9–16 and *Z*11–16:Al in almost opposite ratios may be mainly controlled at one autosomal locus [21], but several QTL associated to the pheromone production in *H. virescens* and *H. subflexa* have been found [48],[49]. As mentioned, the oxidation step is largely unspecific in these species [26],[27], but it is still possible that the oxidases give the final touch to the pheromone depending on the species–specific alcohol profiles. Identifying the oxidase gene(s) involved in moth pheromone biosynthesis together with *in vitro* assays and further candidate gene mapping will be important steps towards a more complete understanding of the genetic basis of sex pheromone production in heliothine moths.

## Supporting Information

Table S1 **Primers used to amplify the partial and full-length sequences of of HvFAR, HsFAR, HarFAR, and HasFAR.** ^*^ The primers pFlHvFARs, pFlHsFARs, and pFlHarFARs, contains an additional Kozak-sequence (small letters) to promote expression efficiency when performing the functional assay. Start codons are emphasized in bold letters. ^1^ GSP used for ORF amplification. ^2^ GSP primer internal for HvFAR, used to amplify the partial HasFAR sequence. ^3^ Degenerate primers for the 3′ region of the pgFARs. ^4^ Primers for RACE amplification. ^5^ GSP for amplification of internal region of HasFAR. ^6^ Primers for sequencing inserts in the pYES2.1 vector.
(XLSX)

Table S2 **Gene tree sequence abbreviations.** Abbreviations for the species from which the reductase sequences used in the gene tree originate.
(XLSX)
